# The risk of intravenous thrombolysis-induced intracranial hemorrhage in Taiwanese patients with unruptured intracranial aneurysm

**DOI:** 10.1371/journal.pone.0180021

**Published:** 2017-06-29

**Authors:** Wei Ting Chiu, Chien Tai Hong, Nai Fang Chi, Chaur Jong Hu, Han Hwa Hu, Lung Chan

**Affiliations:** 1 Department of Neurology, Shuang Ho Hospital, Taipei Medical University, New Taipei City, Taiwan; 2 Department of Neurology, School of Medicine, College of Medicine, Taipei Medical University, Taipei, Taiwan; 3 Stroke Centre, Shuang Ho Hospital, Taipei Medical University, New Taipei City, Taiwan; Universita degli Studi di Palermo, ITALY

## Abstract

**Background:**

The presence of an intracranial aneurysm is contraindicated to recombinant tissue plasminogen activator (r-tPA) treatment for acute ischemic stroke. However, it is difficult to exclude asymptomatic intracranial aneurysms by using conventional, noncontrast head computed tomography (CT), which is the only neuroimaging suggested before r-tPA. Recent case reports and series have shown that administering r-tPA to patients with a pre-existing aneurysm does not increase the bleeding risk. However, Asians are known to have a relatively higher bleeding risk, and little evidence is available regarding the risk of using r-tPA on Asian patients with intracranial aneurysms.

**Methods:**

Medical records from the Shuang Ho hospital stroke registration between July 2010 and December 2014 were retrospectively reviewed, and 144 patients received r-tPA. Unruptured intracranial aneurysms were detected using CT, or magnetic resonance or conventional angiography after r-tPA. The primary and secondary outcomes were the difference in overall intracranial hemorrhage (ICH) and symptomatic ICH after r-tPA. The differences were analyzed using Fisher’s exact or Mann–Whitney U tests, and *p* < 0.05 was defined as the statistical significance.

**Results:**

A total of 144 patients were reviewed, and incidental unruptured intracranial aneurysms were found in 11 of them (7.6%). No significant difference was observed in baseline demographic data between the aneurysm and nonaneurysm groups. Among patients with an unruptured aneurysm, two had giant aneurysms (7.7 and 7.4 mm, respectively). The bleeding risk was not significant different between aneurysm group (2 out of 11, 18%) with nonaneurysm group (7 out of 133, 5.3%) (*p* = 0.14). None of the patients with an unruptured aneurysm had symptomatic ICH, whereas one patient without an aneurysm exhibited symptomatic ICH.

**Conclusions:**

The presence of an unruptured intracranial aneurysm did not significantly increase the risk of overall and symptomatic ICH in Taiwanese patients after they received r-tPA.

## Introduction

Intracranial hemorrhage (ICH) is an unfavorable and potentially fatal complication of thrombolytic therapy based on recombinant tissue plasminogen activator treatment (r-tPA) in patients with acute ischemic stroke [1, 2]. According to guidelines from the Stroke Council of the American Heart Association/American Stroke Association (AHA/ASA), serial exclusion criteria are set to avoid the administration of r-tPA in patients with a high risk of post-r-tPA ICH, such as those with uncontrolled high blood pressure, coagulopathy, and a history of ICH [3, 4]. Patients with intracranial aneurysms are also excluded from receiving r-tPA because r-tPA is suspected to increase the risk of aneurysm rupture and bleeding [5].

The prevalence of intracranial aneurysms is approximately 3.7%–6.6% in the general population globally, most of which are asymptomatic [6, 7]. People are usually unaware of the problem until bleeding, rupture, or incidental detection through cerebral angiography. However, according to the AHA/ASA guidelines, only noncontrast computed tomography (CT) is suggested before the administration of r-tPA, which is unable to exclude intracranial aneurysms. Because the presence of an intracranial aneurysm is contraindicated to r-tPA (and because the earlier the r-tPA is administered, the more favorable is the outcome of recovery), physicians struggle with the dilemma of performing either CT or magnetic resonance (MR) angiography to exclude the possibility of intracranial aneurysms, or shortening the interval between the onset of stroke and the application of r-tPA but omitting this contraindication.

Some case reports have discussed the safety of applying r-tPA to patients with unruptured intracranial aneurysms [8, 9]. In particular, some have demonstrated the bleeding risk of administering r-tPA in patients with unruptured intracranial aneurysm. The risk of overall and symptomatic ICH after receiving r-tPA is identical to that of the nonaneurysm group [10–12]. However, the results from these series do not agree with the guidelines, and physicians still face a similar conflict. Hence, more series from different countries around the world are required. The present study retrospectively analyzed all of the patients with ischemic stroke from a single center in Taiwan who received r-tPA, and addressed the treatment conflict in clinical practice.

## Materials and methods

### Patient selection

This study was approved by the Joint Institutional Review Board of Taipei Medical University (TMU-JIRB) (201503027). Informed consent was waived which was agreed by TMU-JIRB. Medical records of stroke registration between July 2010 and December 2014 from the Shuang Ho hospital, a medical-university-affiliated hospital, were retrospectively reviewed. During this period, 2008 acute cerebral infarction cases were registered, and 146 of them received r-tPA. Intravenous thrombolytic therapy was provided by following the standard protocol adopted from the AHA/ASA guidelines. All patients had received a noncontrast head CT before r-tPA. A history of intracranial aneurysm or any suspicious aneurysm accidentally found during baseline CT was contraindicated to thrombolytic therapy. We excluded two patients because they received intra-arterial thrombolysis after intravenous thrombolysis (bridging therapy). The dosage of intravenous r-tPA thrombolysis was 0.9 mg/kg, and all of the patients received treatment within 3 h of the onset of stroke. Additionally, either head CT with CT angiography or brain MR imaging with MR angiography were performed within 72 h of thrombolysis. Intracranial aneurysms were identified through either CT/MR angiography or further conventional angiography.

Age; sex; National Institutes of Health Stroke Scale (NIHSS) scores before and after r-tPA; and the location, size, and morphology of patients’ aneurysms were recorded. The primary outcome of this study was determining the difference in overall ICH rates following thrombolytic therapy between two groups: patients with and without an intracranial aneurysm. The secondary outcome was understood the difference in symptomatic ICH between the two groups. Symptomatic ICH was defined according to the criteria of the European Cooperative Acute Stroke Study II [13], or by the NIHSS score increasing by more than 4 points. All of the CT/MR and CT/MR/conventional angiography results were analyzed by two independent neurologists.

### Statistical analyses

All analyses were performed using SPSS (version 18.0 edition; SPSS Inc., Chicago, IL, USA) for Windows 10. The continuous variables were presented as mean ± standard deviation, and the categorical variables were calculated as percentages with corresponding 95% confidence intervals. The differences were analyzed using Fisher’s exact test or Mann–Whitney U test as appropriated. A *p* value of <0.05 was considered statistically significant.

## Results

**Fig 1:**
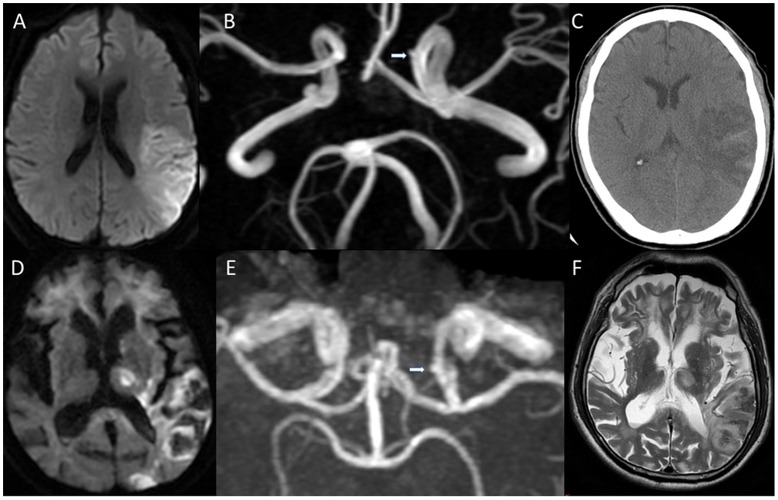
Representative images of patients with intracranial aneurysm with a complicated post-r-tPA intracranial hemorrhage. (A-C) The 72-year-old man had acute ischemic stroke and had received r-tPA within 3 h after the onset of stroke. Follow-up diffusion-weighted imaging (A) (2 h after r-tPA) revealed acute ischemic stroke over the left middle cerebral artery territory. (B) Magnetic resonance angiography revealed a small (2 mm) saccular form of intracranial aneurysm (arrow) over the left internal carotid artery (Source images of magnetic resonance angiography is available in the S1 Fig). (C) Follow-up computed tomography performed 2 days after r-tPA demonstrated petechial hemorrhage in the infarcted margin. (D-F) The 77-year-old woman had acute ischemic stroke and had received r-tPA within 3 h after the onset of stroke. Follow-up diffusion-weighted imaging (D) (2 days after r-tPA) revealed acute ischemic stroke over the left middle cerebral artery territory. (E) Magnetic resonance angiography revealed a small (2.5 mm) saccular form of intracranial aneurysm (arrow) over the left internal carotid artery (Source images of magnetic resonance angiography is available in the S2 Fig). (F) T2-weighted image demonstrated petechial hemorrhage over the infarcted areas without mass effect.

A total of 144 patients who had had a stroke and had received intravenous r-tPA within 3 h were enrolled. All of the patients had received either brain MR imaging and angiography or CT and CT angiography within 72 hours following r-tPA. No significant differences in the baseline clinical characteristics (age, sex, vascular risk factors, smoking, and NIHSS score before r-tPA) were observed, including between the two groups (Table 1). Incidental unruptured intracranial aneurysms were detected in 11 of the patients, and all of the unruptured aneurysms were confirmed by either MR, CT, or conventional angiography (Table 2). Nine unruptured aneurysms were at anterior circulation, and two were at posterior circulation; additionally, two of the patients exhibited a giant unruptured aneurysm (diameter ≥ 7 mm). Two (18.2%) of the patients with an unruptured aneurysm also had ICH, and the diameter of their aneurysms was 2.5 and 2 mm. Notably, the ICH episodes that occurred in the aneurysm group were not related to the location of aneurysm (Fig 1 and S1 and S2 Figs), and neither of these patients had symptomatic ICH. In the nonaneurysm group, seven out of the 133 patients (5.3%) had ICH and one had symptomatic ICH (Table 3). No significant difference in ICH and symptomatic ICH risk was observed between the aneurysm and nonaneurysm groups (both *p* > 0.05) (Table 4). The presence of unruptured intracranial aneurysm was not correlated with overall and symptomatic ICH (S1 Table).

**Table 1:** Demographic data of acute ischemic stroke patients with and without an aneurysm.

	Aneurysm (+)	Aneurysm (−)	*p* value
Case number	11	133	
Age (years)	74.8 ± 11.8	69.5 ± 12.1	0.14
Female sex	5	77	0.53
Smoking	1	32	0.46
Atrial fibrillation	5	37	0.30
Coronary artery disease	2	18	0.65
Hypertension	8	103	0.71
Diabetes mellitus	1	40	0.18
Hyperlipidemia	6	59	0.54
NIHSS score before r-tPA	11.9 ± 4.8	14.0 ± 6.4	0.28

Note: NIHSS, National Institute Health Stroke Scale; r-tPA, recombinant tissue plasminogen activator treatment

**Table 2:** List of patients with intracranial aneurysm who received r-tPA.

Age (years)	Sex	NIHSS before r-tPA	Vascular territory of stroke	Morphology of aneurysm	Location of aneurysm	Size of aneurysm (mm)	ICH
53	M	9	R’t MCA	Saccular	Ant. com. a.	3	N
81	M	15	R’t PCA	Saccular	BA	3	N
86	F	8	R’t MCA	Saccular	R’t MCA	5	N
90	F	9	R’t MCA	Saccular	R’t ICA	2.6	N
83	F	15	BA	Fusiform	R’t ICA	3.1	N
77	F	24	L’t MCA	Saccular	L’t ICA	2.5	Y
79	F	7	L’t ACA	Saccular	L’t ICA	3	N
67	F	12	R’t MCA	Saccular	L’t ICA	7.4	N
72	M	11	L’t MCA	Saccular	L’t ICA	2	Y
79	M	9	BA	Saccular	R’t MCA	7.7	N
56	M	12	L’t MCA	Saccular	L’t MCA	2.5	N

Note: M, male; F, female; NIHSS, National Institutes of Health Stroke Scale; r-tPA, recombinant tissue plasminogen activator treatment; MCA, middle cerebral artery; PCA, posterior cerebral artery; BA, basilar artery; ACA, anterior cerebral artery; Ant. com. a., anterior communicating artery; ICA, internal carotid artery; ICH, intracranial hemorrhage.

**Table 3:** Clinical information about the patients with ICH after r-tPA treatment.

Patients of ICH	Aneurysm (location/size)	Location of stroke	Location of ICH	Type of ICH (ECASS-II)[14]	Management of ICH
1	N	R't BG	R't BG	PH1	M
2	N	R’t P	R’t P	HI2	M
3	N	R’t T-P	R’t T-P	HI2	M
4	N	L’t F-T-P	L’t F-T-P	HI1	M
5	N	R’t BG	Bil F	PH1	M
6	N	R't F	R’t F-P	PH2	S
7	N	R't F	R't F	HI2	M
8	Y (L’t ICA/2.5mm)	L't O-P	L't O-P	HI2	M
9	Y (L’t ICA/2mm)	L't P	L't P	HI1	M

Note: BG, basal ganglia; ECASS-II, European Cooperative Acute Stroke Study II (hemorrhagic infarction types 1 and 2 (HI1 and HI2) and parenchymal hematoma types 1 and 2 (PH1 and PH2)); F, frontal lobe; ICA, internal carotid artery; ICH, intracranial hemorrhage, M, medical treatment; O, occipital lobe; P, parietal lobe; S, surgical treatment.

**Table 4:** Comparison of overall intracranial hemorrhage (ICH) and symptomatic ICH between patients with/without aneurysm.

	Aneurysm (-)	Aneurysm (+)	p-value
Without ICH	126	9	
Overall ICH	7	2	0.09
Symptomatic ICH	1	0	1.00

## Discussion

The present study demonstrated that the presence of an unruptured intracranial aneurysm did not significantly increase the risk of overall and symptomatic ICH in Taiwanese patients with acute ischemic stroke after they had received r-tPA. Among the patients with complicated post-thrombolytic ICH, the location of their unruptured aneurysm was not associated with ICH. Therefore, the results hinted that the presence of an intracranial aneurysm may not require exclusion from intravenous thrombolytic treatment.

A giant aneurysm (diameter ≥ 7 mm), posterior circulation location, female sex, and smoking are associated with a higher risk of cerebral aneurysm rupture [9]. Thrombolytic agents alter the vascular permeability of the vascular basal lamina and the conformation of blood vessel endothelium and basilar membrane, which can weaken the defective vascular wall of an aneurysm and thus induce rupture and hemorrhage [15, 16]. Based on this concern, the presence of an intracranial aneurysm contraindicates thrombolytic therapy for acute ischemic stroke [13, 17]. Practically, however, exclusion on the basis of a silent intracranial aneurysm before administering r-tPA is difficult. First, in the general population, the prevalence of intracranial aneurysms is approximately 3.7%–6.6%. Patients with a history of hypertension are more likely to have intracranial aneurysms and are also at a higher risk of ischemic stroke. However, most of these unruptured aneurysms are asymptomatic and people are unaware of them. Second, the therapeutic interval between the onset of stroke and thrombolytic r-tPA for acute ischemic stroke is extremely short. To shorten the interval between the onset of stroke and initiation of treatment, noncontrast conventional head CT is the only suggested neuroimaging study, and detection of an intracranial aneurysm through CT is nearly impossible. Although contrast CT or MR imaging may be able to detect unruptured aneurysms, these methods require more time than noncontrast CT and may delay the treatment. Due to these limitations, excluding all asymptomatic intracranial aneurysms before the administration of r-tPA is nearly impossible.

Since r-tPA was approved for acute ischemic stroke, several case reports have demonstrated the effects of r-tPA on patients with asymptomatic cerebral aneurysms, which were detected during follow-up neuroimaging [18–24]. Some of these cases did not involve the complication of ICH during aneurysm rupture; other case series investigated the safety of r-tPA for patients with unruptured cerebral aneurysms, and noted that the asymptomatic and incidentally detected unruptured cerebral aneurysms did not increase the risk of post-r-tPA ICH [10–12, 25, 26]. However, most of the aforementioned studies were conducted in Western countries and included limited Asians, who tend to exhibit a higher risk of post-r-tPA ICH because of coagulation differences [27–29]. The present study, conducted in Taiwan, partially addresses this gap. Specifically, we found that the risk of post-r-tPA symptomatic ICH did not increase among the patients with intracranial aneurysms; moreover, in the patients with ICH, the location of their hemorrhage was not relevant to the aneurysm. These data indicate that even in Asians, incidental or asymptomatic cerebral aneurysms are safe for thrombolytic r-tPA at the onset of acute ischemic stroke. Taking these series and case reports together, we suggest that the presence of an unruptured intracranial aneurysm is not a contraindication of administering r-tPA for acute ischemic stroke.

The present study had some limitations. First, not all the cerebral aneurysms were detected using conventional angiography. The sensitivity of MR/CT angiography in detecting cerebral aneurysm is inferior to that of the conventional angiography, particularly for detecting small-sized aneurysms, and therefore, the overall number of aneurysms may be underestimated and their classifications may be biased. Although, smaller-sized (< 7 mm in diameter) aneurysms are less likely to rupture and cause bleeding, ICH may mask the aneurysm. Rupture and bleeding of aneurysms may also induce vasospasm and transient disappearance of the aneurysm, and ICH may lead to the underestimation of the aneurysm-related bleeding.

In conclusion, the present study demonstrated that incidental unruptured intracranial aneurysms did not increase the risk of ICH, particularly symptomatic ICH, after r-tPA for ischemic stroke in Taiwanese patients. To shorten the interval of the onset of stroke and the administration of r-tPA, and to avoid guideline violations by physicians, an amendment of the contraindications of r-tPA for patients with intracranial aneurysms should be considered.

## Supporting information

S1 Fig Image of a 72 years old man, post r-tPA ICH with unruptured aneurysm. (TIF)

S2 Fig Image of a 77 years old woman, post r-tPA ICH with unruptured aneurysm. (TIF)

S1 Table The correlation between the present of unruptured cerebral aneurysm and post r-tPA ICH. (PDF)
