# Supplementary figures and images for: The risk of intravenous thrombolysis-induced intracranial hemorrhage in Taiwanese patients with unruptured intracranial aneurysm
Source: PLoS One. 2017 Jun 29;12(6):e0180021. doi: 10.1371/journal.pone.0180021 (PMC5491104; doi:10.1371/journal.pone.0180021)

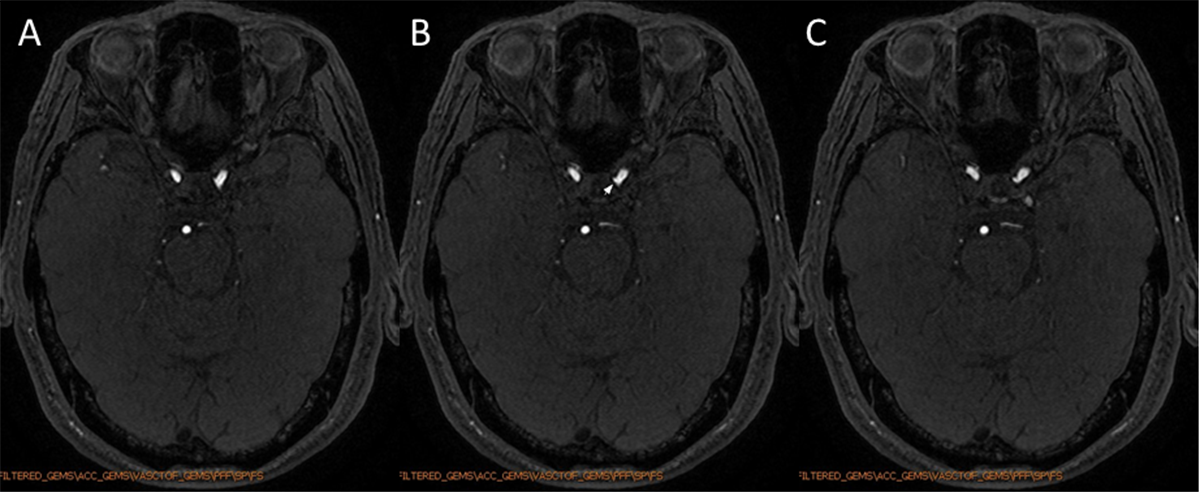

Supplement: S1 Fig — (TIF) [file pone.0180021.s001.tif]

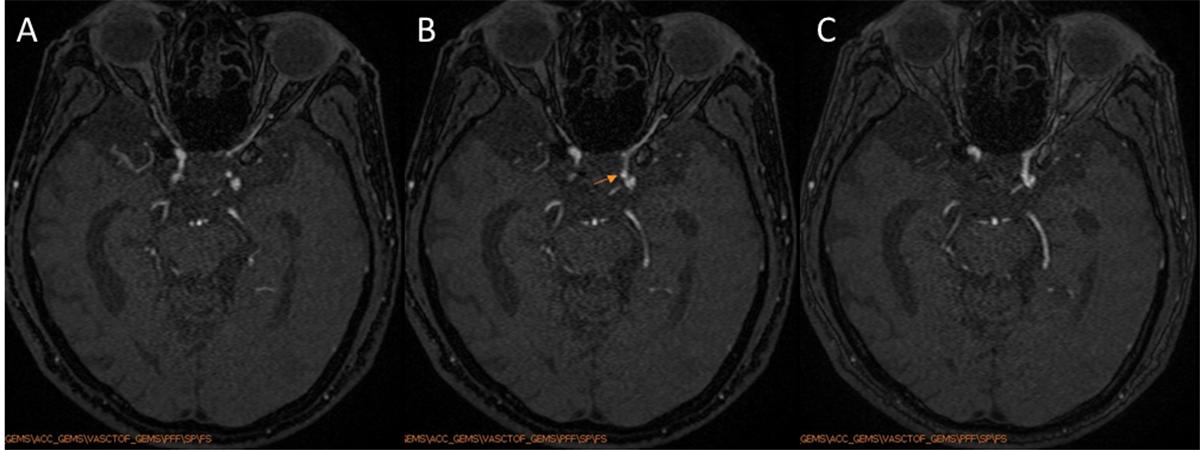

Supplement: S2 Fig — (TIF) [file pone.0180021.s002.tif]
