# Supplementary material for: The risk of intravenous thrombolysis-induced intracranial hemorrhage in Taiwanese patients with unruptured intracranial aneurysm
Source: PLoS One. 2017 Jun 29;12(6):e0180021. doi: 10.1371/journal.pone.0180021 (PMC5491104; doi:10.1371/journal.pone.0180021)
Supplement: S1 Table — (PDF) [file pone.0180021.s003.pdf]

Supplementary Table

The correlation between the present of unruptured cerebral aneurysm and ICH among patients with ischemic stroke received r-tPA treatment

		Sex	Age	NIHSS	ICH	sICH	Aneurysm
Sex	*r*	1	-.123	-.128	.163	.072	-.018
	p-value		.142	.125	.051	.393	.830
Age (y/o)	*r*	-.123	1	.111	.009	-.063	.141
	p-value	.142		.187	.913	.453	.091
NIHSS	*r*	-.128	.111	1	-.021	-.100	-.090
	p-value	.125	.187		.803	.234	.281
ICH	*r*	.163	.009	-.021	1	.324**	.142
	p-value	.051	.913	.803		.000	.090
sICH	*r*	.072	-.063	-.100	.324**	1	-.024
	p-value	.393	.453	.234	.000		.775
Aneurysm	*r*	-.018	.141	-.090	.142	-.024	1
	p-value	.830	.091	.281	.090	.775	

Abbreviations: ICH, intracranial hemorrhage; sICH, symptomatic intracranial hemorrhage; NIHSS: national institute of health stroke scale;
